# Urinary Matrix Metalloproteinase-7 and Prediction of AKI Progression Post Cardiac Surgery

**DOI:** 10.1155/2019/9217571

**Published:** 2019-11-19

**Authors:** Fan Fang, Weihong Luo, Manqiu Yang, Peiliang Yang, Xiaobing Yang

**Affiliations:** ^1^Division of Nephrology, Nanfang Hospital, Southern Medical University, National Clinical Research Center for Kidney Disease, State Key Laboratory of Organ Failure Research, Guangdong Institute, Guangzhou, China; ^2^Department of Nephrology, Zhongshan Hospital, Xiamen University, Xiamen, China

## Abstract

**Aims:**

Early detection of patients at high risk for progressive acute kidney injury (AKI) after cardiac surgery remains a major challenge. We aim to evaluate the utility of urinary matrix metalloproteinase-7 (uMMP-7) and other reported biomarkers for predicting AKI progression during postoperative hospital stay.

**Methods:**

We conducted a prospective, multicenter cohort study in 121 adult patients with stage 1 or 2 AKI after cardiac surgery. uMMP-7 and other well-reported biomarkers (uIL-18, uNGAL, and UACR) were measured at time of AKI clinical diagnosis. The primary outcome is the progression of AKI after cardiac surgery, defined as worsening of AKI stage (stage 1 to either stage 2 or stage 3 or from stage 2 to stage 3).

**Results:**

A level of uMMP‐7 > 7.8 *μ*g/g Cr at time of AKI diagnosis conveyed an 8-fold risk of AKI progression as compared to those with uMMP‐7 < 2.7 *μ*g/g after adjusting for clinical risk factors. The performance of uMMP-7 for predicting progressive AKI was good with an AUC of 0.80. The combination of uMMP-7 and IL-18 produces the greatest AUC for predicting progressive AKI. Addition of uMMP-7 to the clinical risk factor model significantly improved risk reclassification for AKI progression.

**Conclusions:**

uMMP-7, measured at time of AKI clinical diagnosis, is a novel biomarker for predicting the progression of AKI after cardiac surgery. Adding uMMP-7 to the clinical risk factor model may be used as a noninvasive approach to identify a subpopulation that is at high risk for progressive AKI after cardiac surgery.

## 1. Introduction

Acute kidney injury (AKI) is common in patients who are receiving cardiac surgery by cardiopulmonary bypass, with high morbidity (ranging 30-40%) [1] and mortality (ranging 10-20%) [2]. The episodes and severity of AKI were linked to all kinds of poor outcomes and increased risk of future chronic kidney disease (CKD) and even end-stage kidney disease [3].

Most patients who develop AKI after cardiac surgery experience a mild form of AKI (e.g., Kidney Disease Improving Global Outcomes (KDIGO) stage 1). However, about 10%–15% of patients with initial mild AKI after surgery will progress to a more severe stage (KDIGO stage 2 or 3) or even require dialysis during their postoperative hospital stay [4, 5]. Recent studies have consistently shown that risk of mortality exponentially increased with increasing stages of AKI [6, 7]. Early identification of patients at high risk for progressive AKI after cardiac surgery would help physicians to improve monitoring and care of postoperative patients, guide patient counseling and decision-making, and facilitate participation in interventional trials of AKI [5].

During the past decade, there has been increasing interest on searching new biomarkers for AKI development and prognosis in the setting of cardiac surgery. Several biomarkers, such as NGAL (neutrophil gelatinase-associated lipocalin) and IL-18 (interleukin-18), were found to predict AKI development before the clinical diagnosis was reached [8]. Furthermore, the biomarker level can also predict in-hospital outcomes, such as receiving renal replacement therapy, long hospital stays, and in-hospital mortality. A few studies also showed that biomarkers measured at the early stage of established AKI predicted the progression of AKI after cardiac surgery but yielded modest performance in general [4, 5].

Matrix metalloproteinase-7 (MMP-7) is one of the smallest secreted matrix metalloproteinases, predominantly localized in renal tubular epithelium, and can be easily excreted into urine [9]. Little or no MMP-7 expression is detected in the normal kidney. However, its expression is markedly induced in human and animal models of kidney injury, which is primary controlled transcriptionally by *β*-catenin, the principal downstream mediator of canonical Wnt signaling [10]. We have previously shown that urinary matrix metalloproteinase-7 (uMMP-7) levels faithfully reflect renal Wnt/*β*-catenin activity, and the signal pathway is activated in AKI induced by ischemia-reperfusion injury or renal toxicity [10, 11].

Based on the above basic finding, we have recently validated that the postoperative uMMP-7 level predicts subsequent development of AKI and poor outcomes in patients who receive cardiac surgery [9]. Whether uMMP-7 can also serve as a biomarker forecasting the progression of AKI remains unknown. We therefore hypothesize that uMMP-7 might serve as a biomarker for predicting the progression of AKI which developed after cardiac surgery, and the combination of uMMP-7 and other reported biomarkers can further improve the prediction for AKI progression after cardiac surgery.

Here, we conducted a prospective, multicenter cohort study in 121 adult patients with stage 1 or 2 AKI after cardiac surgery to evaluate the utility of uMMP-7 and other reported biomarkers for predicting AKI progression during postoperative hospital stay.

## 2. Methods

### 2.1. Patients and Study Design

As previously described [9], we prospectively enrolled 398 adult patients who undergo elective cardiac surgery at six academic medical centers in China between September of 2013 and September of 2014. Of all 398 patients, only patients who developed initial stage 1 or stage 2 AKI (KDIGO criteria 2012 [12]) and with all tested biomarkers available at time of initial AKI diagnosis were included in this study. Patients were excluded if their initial AKI diagnosis was stage 3 since they would not progress further. The study was approved by the Institute Review Board of the National Clinical Research Center for Kidney Disease, and all participants provided written informed consent.

### 2.2. Procedure

We collected spot urine and blood samples before operation and at frequent intervals for 5 days after surgery. Urine sample collection and processing have been previously reported [9]. uMMP-7 and other reported biomarkers (urinary NGAL (uNGAL), urinary IL-18 (uIL-18)), as well as the urinary albumin to creatinine ratio (UACR), were measured at the day of initial AKI diagnosis. Serum creatinine was measured at least daily post operation and recorded for every patient throughout the hospital stay.

### 2.3. Biomarker Measurement

All of the biomarkers were measured in our central lab using a standard protocol to reduce the intra- or interassay variability. All the samples were labeled using study identification numbers without personal identifiers or clinical conditions. Urinary and MMP-7 levels were measured by an ELISA kit (DMP700; R&D Systems, Minneapolis, MN) according to the manufacturer's instructions. Other reported biomarkers of renal injury, such as uNGAL, uIL-18, and UACR, were measured by available commercial ELISA kits according to the manufacturer's instruction.

### 2.4. Outcome Definitions

The primary outcome was the progression of AKI, defined as worsening of the KDIGO stage (from stage 1 to either stage 2 or stage 3 or from stage 2 to stage 3). Patients treated with acute dialysis at any point during hospitalization were defined as stage 3. Additional clinical outcomes were needed for acute dialysis, in-hospital mortality. Patients who died without progression were excluded from the primary analysis because death may have been a competing risk for progression for these patients.

### 2.5. Statistical Analyses

We performed the analyses with SPSS software (version 17.0). To compare continuous variables, we used a two-sample *t*-test or a Mann–Whitney *U* test. To compare categorical variables, we used the chi-squared or Fisher exact test. All tests were two-tailed, and *P* < 0.05 was considered significant.

As previously described [7], we categorized uMMP-7 tertile essentially as a continuous variable and then performed logistic regression on created variables. We determined the adjusted odds ratios (OR) of AKI progression with multiple logistic regression analysis. Due to the limited events, we adjusted for major risk factors for AKI progression after surgery, i.e., baseline estimated GFR, preoperative NYHA class, and cardiopulmonary bypass time, as well as percent change in serum creatinine from the baseline to the time of initial AKI diagnosis. uMMP-7 was also modeled as a continuous variable (log-transformed).

To compare the performance of uMMP-7 and other biomarkers, a conventional area under the receiver-operating characteristic (ROC) curve (AUC) was generated. To evaluate the utility of the biomarkers on risk classification, we determined the category-free net reclassification improvement (NRI) and the integrated discrimination improvement (IDI), as previously described [13, 14].

The performance of uMMP-7 for predicting AKI progression was internally validated by a bootstrap method with 1000 replications [15].

## 3. Results

### 3.1. Cohort Characteristics

A total of 121 patients who developed initial stage 1 or 2 AKI after cardiac surgery and with all tested biomarkers available at time of AKI were included in the final analysis.

Among 121 patients with AKI, 116 (95.8%) patients developed AKI within 72 hours post operation. A total of 28 patients (23.1%) progressed to a higher stage of AKI during their postoperative hospital stay (20 individuals progressed to stage 2 and 8 patients progressed to stage 3), 5 of 28 (17.9%) progressors received acute dialysis; 4 of 28 (14.3%) had AKI progression and subsequently died during their hospitalization, 93 patients (76.9%) persisted in stage 1 or 2 AKI, and none of them died or received acute dialysis during their postoperative hospital stay.

Table 1 shows the preoperative characteristics of 121 patients who developed or did not develop progressive AKI after cardiac surgery. There was no statistical difference in proportion of patients using RAS inhibitors or diuretics before surgery between those with or without AKI progression. Compared with those without AKI progression, patients with progressive AKI had lower preoperative eGFR and longer CPB time.

Table 2 compares postoperative characteristic and outcomes of patients with or without AKI progression. Patients with progressive AKI had a higher serum creatinine levels on the day of AKI diagnosis as compared to those without progressive AKI. Change of serum creatinine from the baseline (preoperative level) at the time of AKI diagnosis was also greater in patients with AKI progression. Levels of uMMP-7 and 3 previously reported urinary biomarkers (uIL-18, uNGAL, and UACR) were significantly higher in patients with progressive AKI as compared to those without. Patients with AKI progression had more adverse outcomes, such as receiving acute dialysis and in-hospital death, as compared with those without AKI progression (Table 2).

### 3.2. The Performance of uMMP-7 and Other Urinary Biomarkers for Predicting the Progression of AKI

The median of uMMP-7 and other biomarkers were significantly higher in patients with AKI progression compared to nonprogressors. There were graded responses across the tertiles of uMMP-7 level and the risk of AKI progression in the univariate model and remained statistically significant after adjusting for major clinical risk factors (Table 3). In the adjusted model, patients with the highest tertile of uMMP-7 (>7.8 *μ*g/g Cr) had a 7.8-fold higher risk of AKI progression as compared with those with the lowest tertile of uMMP-7 (<2.7 *μ*g/g Cr). When uMMP-7 was modeled as a continuous variable, higher levels of uMMP-7 were also associated with increased risk of progressive AKI in a multivariate model (OR per SD, 3.0; 95% CI, 1.4-6.2, *P* = 0.002).

**Figure 1:**
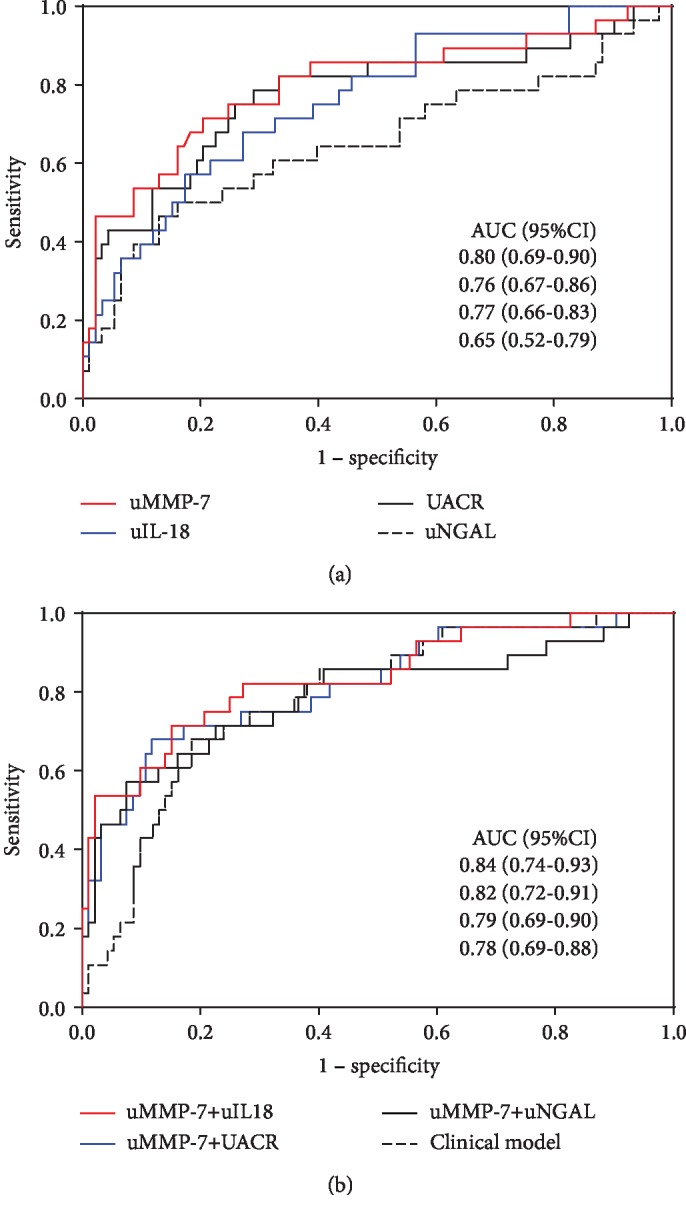
ROC analyses for predicting AKI progression. (a) The AUCs of urinary biomarkers (uMMP-7, uIL-18, UACR, and uNGAL), at the time of AKI diagnosis, for predicting AKI progression. (b) The performance of combination of urinary biomarkers, and clinical model alone, for predicting AKI progression.

uMMP-7 presented good performance for predicting progressive AKI after surgery, with an AUC of 0.80, greater than those of well-reported biomarkers (uIL-18, AUC 0.76; UACR, AUC 0.77; and uNGAL, AUC 0.65) (Figure 1(a)). The combination of uMMP-7 with uL-18 or UACR further improved the performance for predicting the progression of AKI, with AUCs of 0.84 (uMMP-7 and uIL-18) and 0.82 (uMMP-7 and UACR) (Figure 1(b)).

The performance of uMMP-7 was further confirmed by the bootstrap internal validation, in which the average AUC for predicting AKI progression (0.80; 95% CI 0.78-0.81) was comparable to that in the test cohort. Using the raw data without urinary creatinine correction, uMMP-7 also presented a comparable AUC (0.79, 95% CI 0.68-0.88) for predicting AKI progression.

### 3.3. The Improvement of the Risk Classification with the Injury Biomarkers to the Clinical Model

The addition of uMMP-7 to the clinical risk factor model significantly improved risk classification for AKI progression, as evidenced by the net reclassification index (NRI) and the integrated discrimination improvement (IDI). Compared to reported biomarkers, uMMP-7 improved category-free NRI of 0.92, which was the greatest among those of all tested biomarkers (Table 4).

## 4. Discussion

In this prospective, multicenter study of adult patients who undergone cardiac surgery, we firstly showed that uMMP-7, measured at time of AKI diagnosis, is a novel biomarker for predicting the progression of AKI. A level of uMMP‐7 > 7.8 *μ*g/g Cr at time of AKI diagnosis denoted an 8-fold risk of AKI progression as compared to those with uMMP‐7 < 2.7 *μ*g/g after adjusting for major clinical risk factors. The performance of uMMP-7 for predicting progressive AKI post operation was good with an AUC of 0.80. The combination of uMMP-7 and IL-18 produced the greatest AUC for predicting progressive AKI. The addition of uMMP-7 to the clinical risk factor model significantly improved risk reclassification for AKI progression after cardiac surgery.

In recent years, there were studies demonstrated that renal injury biomarkers can detect acute kidney injury after cardiac surgery earlier than the increase of serum creatinine [8, 9]. Several biomarkers, such as IGF binding protein 7 and tissue inhibitors of metalloproteinase, have been approved by the US Food and Drug Administration as a first-of-a-kind test to help determine if surgical patients are at risk of developing AKI [16]. However, the identification of biomarkers that predict AKI progression in patients with established AKI has not been fully highlighted [8]. AKI progression after cardiac surgery is associated with increased risk of poor outcomes. In our cohort, patients who initially developed mild AKI and progressed to higher stages had mortality of 14% versus 0% in those who presented in original stages but not progressed, consistent with the previous report [5]. It is therefore critical to identify patients at highest risk of AKI progression so as to guide prognosis and management decisions. There are several studies that reported that biomarkers, measured at time of AKI clinical diagnosis, predicted AKI development after cardiac surgery [4, 5]. A recent study tested the ability of 32 biomarkers to predict worsening of renal function in patients with AKIN stage 1 AKI after cardiac surgery [4]. They found that uIL-18 was the best predictor of worsening AKI. In a larger study from the Translational Research Investigating Biomarker Endpoints-AKI consortium, uIL-18, UACR, and uNGAL measurement at the time of AKI diagnosis predicted the progression of AKI in adults after cardiac surgery [5]. In our study, elevation of uMMP-7 is an independent predictor of progressive AKI after cardiac surgery after adjusting for major preoperative and intraoperative risk factors and provides good performance for predicting AKI progression. Furthermore, adding uMMP-7 to the clinical risk model significantly improves risk reclassification for AKI progression, suggesting that early measurement of uMMP-7 at time of AKI might be helpful to accurately identify patients at increased risk for AKI progression, and may offer clinicians an earlier time window to halt or reverse ongoing kidney injury.

The potential role of elevated renal MMP-7 in human AKI progression is waiting for exploration. uMMP-7 is a marker faithfully reflecting intrarenal Wnt/beta-catenin activity and dependably mirrors its expression in renal parenchyma, particularly in the tubular epithelium [10]. Tubular MMP-7 expression is significantly induced after renal ischemia-reperfusion injury [17]. Recent experimental data found that sustained Wnt/beta-catenin activating after ischemia-reperfusion injury might drive kidney injury progression [11], suggesting that uMMP-7 could be selected as a marker of AKI progression.

To further enhance the ability of biomarkers for predicting AKI progression after cardiac surgery, carefully selecting and combining biomarkers might be a better approach for greater use. Urinary IL-18, an inflammation marker of injury, has been consistently reported as a predictive biomarker for progressive AKI after cardiac surgery [4, 5]. In our study, combining uIL-18 and uMMP-7 produced the greatest AUC (0.84) compared with combining uNGAL or UACR, supporting a multibiomarker approach which might further improve the predictive ability of biomarker for AKI progression after cardiac surgery [8].

Our study has the following strengths. First, it is a multicenter, prospective cohort study and relied on standardized AKI staging criteria (KDIGO) that are currently used in the international renal community. Second, serum creatinine was measured everyday post cardiac surgery, which allowed us to precisely define AKI and determine AKI progression. Third, we simultaneously measured previously reported biomarkers and assessed the predictive performance of uMMP-7 with other established biomarker for predicting AKI progression in the setting of cardiac surgery, which directly compares the predictive ability of biomarkers alone or in combination. This study also had limitations. Urinary creatinine excretion is not at a steady state during AKI; 24 h urinary excretion of MMP7 would be more meaningful. The number of primary outcome was relatively small, and all patients were Chinese adults; validation studies from other ethnic populations are warranted.

In conclusion, uMMP-7 measured at time of AKI clinical diagnosis predicts AKI progression. Adding uMMP-7 to the clinical risk factor model may be used as a noninvasive approach to identify patients that are at high risk for progressive AKI after cardiac surgery, which may facilitate patient counseling and optimize management in the setting of cardiac surgery.

## Figures and Tables

**Table 1:** Preoperative characteristics of patients with and without AKI progression^a^.

Variables	AKI progression		*P*
	Yes (*n* = 28)	No (*n* = 93)	
Age (y)	49.3 ± 10.9	51.2 ± 11.2	0.42
Male, *n* (%)	20 (71.4)	44 (47.3)	0.03
Diabetes, *n* (%)	2 (7.1)	7 (7.5)	0.99
Hypertension, *n* (%)	8 (28.6)	22 (23.7)	0.62
Congestive heart failure, *n* (%)	10 (35.7)	50 (53.8)	0.13
Preoperative NYHA class III or IV	5 (17.9)	21 (22.6)	0.78
Preoperative creatinine (*μ*mol/L)	102.1 ± 40.3	80.3 ± 20.8	<0.001
Preoperative eGFR (mL/min/1.73 m^2^)^b^	76.1 ± 25.3	86.7 ± 18.9	0.02
Preoperative medication, *n* (%)			
RAS inhibitors	12 (42.9)	23 (24.7)	0.09
Diuretics	25 (89.3)	89 (95.7)	0.35
Operative variables			
CABG alone, *n* (%)	1 (3.5)	2 (2.1)	0.55
Valve alone, *n* (%)	20 (71.4)	66 (70.9)	0.99
CABG and valve surgery, *n* (%)	2 (7.1)	5 (5.3)	0.66
CPB time (min)	163.6 ± 60.2	131.4 ± 51.8	0.006
Cross clamp time (min)	90.5 ± 40.6	86.3 ± 33.5	0.59

^a^AKI progression is defined as worsening of the AKI stage (stage 1 to either stage 2 or stage 3 or from stage 2 to stage 3). ^b^Calculated by CKD-Epidemiology Collaboration equation 2009. Abbreviation: NYHA: New York Heart Association; eGFR: estimated glomerular filtration rate; RAS: renin-angiotensin system; CABG: coronary artery bypass grafting; CPB: cardiopulmonary bypass.

**Table 2:** Postoperative characteristics and outcomes of patients with and without AKI progression^a^.

	AKI progression		*P*
	Yes (*n* = 28)	No (*n* = 93)	
Time of AKI			
Within 3 days after surgery, *n* (%)	28 (100.0)	88 (94.6)	0.59
SCr on the day of AKI diagnosis (*μ*mol/L)	184.2 ± 86.2	127.6 ± 35.9	<0.001
Change of SCr on the day of AKI (*μ*mol/L)^b^	82.1 ± 79.1	46.8 ± 26.8	<0.001
Change of SCr on the day of AKI (%)^c^	90.7 ± 89.1	60.8 ± 35.4	0.01
Biomarkers on the day of AKI diagnosis			
uMMP-7 (*μ*g/g Cr)	10.4 (5.6-25.7)	3.3 (1.3-7.3)	<0.001
uIL-18 (ng/g Cr)	305.5 (155.7-596.0)	110.9 (32.9-232.0)	<0.001
uNGAL (*μ*g/g Cr)	146.6 (31.4-391.9)	50.8 (19.8-113.6)	0.01
UACR (mg/g Cr)	163.8 (84.1-312.9)	39.8 (16.2-100.7)	<0.001
Outcomes			
Acute dialysis, *n* (%)	5 (17.9)	0 (0.0)	<0.001
In-hospital death, *n* (%)	4 (14.3)	0 (0.0)	0.002

^a^AKI progression is defined as worsening of the AKI stage (stage 1 to either stage 2 or stage 3 or from stage 2 to stage 3). ^b^Serum creatinine level on the day of AKI diagnosis minus baseline serum creatinine level. ^c^(SCr level on the day of AKI diagnosis‐baseline SCr level)/baseline SCr level^∗^ 100%. Abbreviation: SCr: serum creatinine; uMMP-7: urinary matrix metalloproteinase-7; uIL-18: urinary interleukin-18; uNGAL: urinary neutrophil gelatinase-associated lipocalin; UACR: urinary albumin to creatinine ratio.

**Table 3:** Multivariate logistic regression analyses of uMMP-7 for predicting AKI progression^a^.

uMMP-7	Cut points (*μ*g/g Cr)	Progression (%)	Unadjusted OR (95% CI)	*P*	Adjusted OR^b^ (95% CI)	*P*
*Categorical*						
Low (Tertile 1, *n* = 40)	<2.7	10.0	1.0 (referent)		1.0 (referent)	
Medium (Tertile 2, *n* = 41)	2.7-7.8	12.2	1.3 (0.3-5.0)	0.75	0.8 (0.2-4.2)	0.72
High (Tertile 3, *n* = 40)	>7.8	47.5	8.1 (2.4-27.2)	0.001	7.8 (1.9-36.0)	0.003
*Continuous*						
Per SD increase for lg transform	—	—	3.9 (1.8-8.2)	<0.001	3.0 (1.4-6.2)	0.002

^a^AKI progression is defined as worsening of the AKI stage (stage 1 to either stage 2 or stage 3 or from stage 2 to stage 3). ^b^Adjusted for preoperative eGFR, preoperative NYHA class, CPB time, and change in postoperative serum creatinine from baseline at the time of AKI diagnosis.

**Table 4:** Risk reclassification of adding uMMP-7 and other biomarkers to the clinical model for predicting AKI progression^a^.

Variables	Category-free NRI (95% CI)	*P*	Category-free NRI (95% CI)				IDI (95% CI)	*P*
			With events	*P*	Without events	*P*		
Clinical risk factors^b^	Referent		Referent		Referent		Referent	
Clinical risk factors+uMMP-7	0.92 (0.60-1.20)	<0.001	0.57 (0.25-0.90)	0.001	0.35 (0.15-0.54)	0.001	0.20 (0.12-0.28)	<0.001
Clinical risk factors+uIL-18	0.88 (0.50-1.16)	<0.001	0.50 (0.16-0.84)	0.006	0.38 (0.19-0.58)	<0.001	0.18 (0.09-0.27)	<0.001
Clinical risk factors+uNGAL	0.67 (0.47-0.87)	0.01	0.43 (0.07-0.78)	0.02	0.24 (0.04-0.44)	0.02	0.08 (0.02-0.14)	0.001
Clinical risk factors+UACR	0.88 (0.50-1.16)	<0.001	0.48 (0.25-0.80)	0.007	0.40 (0.21-0.62)	<0.001	0.18 (0.09-0.27)	<0.001

^a^AKI progression is defined as worsening of the AKI stage (stage 1 to either stage 2 or stage 3 or from stage 2 to stage 3). ^b^Comprised of preoperative eGFR, CPB time, and change in postoperative serum creatinine from baseline at the time of AKI diagnosis. Abbreviation: NRI: net reclassification improvement; IDI: integrated discrimination improvement; CI: confidence interval.

## Data Availability

The data used to support the findings of this study are available from the corresponding author upon request.
